# Proteomic Analysis of Skeletal Muscle and White Adipose Tissue after Aerobic Exercise Training in High Fat Diet Induced Obese Mice

**DOI:** 10.3390/ijms24065743

**Published:** 2023-03-17

**Authors:** Tzu-Jung Chou, Chia-Wen Lu, Li-Yu Lin, Yi-Ju Hsu, Chi-Chang Huang, Kuo-Chin Huang

**Affiliations:** 1 Department of Family Medicine, National Taiwan University Hospital Hsin-Chu Branch, Hsinchu 300, Taiwan; joycechou0307@gmail.com; 2 Graduate Institute of Clinical Medicine, National Taiwan University College of Medicine, Taipei 100, Taiwan; biopsycosocial@gmail.com; 3 Department of Family Medicine, National Taiwan University Hospital, Taipei 100, Taiwan; 4 Department of Family Medicine, National Taiwan University College of Medicine, Taipei 100, Taiwan; 5 Graduate Institute of Sports Science, National Taiwan Sport University, Taoyuan 333, Taiwan; liyuphdntsu0913@gmail.com (L.-Y.L.); ruby780202@ntsu.edu.tw (Y.-J.H.); 6 Graduate Institute of Physiology, National Taiwan University College of Medicine, Taipei 100, Taiwan

**Keywords:** aerobic exercise, ingenuity pathways analysis, obesity, proteome

## Abstract

Obesity is associated with excessive fat accumulation in adipose tissue and other organs, such as skeletal muscle, whereas aerobic exercise (AE) plays an important role in managing obesity through profound protein regulation. Our study aimed to investigate the impact of AE on proteomic changes in both the skeletal muscle and the epididymal fat pad (EFP) of high-fat-diet-induced obese mice. Bioinformatic analyses were performed on differentially regulated proteins using gene ontology enrichment analysis and ingenuity pathway analysis. Eight weeks of AE significantly reduced body weight, increased the serum FNDC5 level, and improved the homeostatic model assessment of insulin resistance. A high-fat diet caused alterations in a subset of proteins involved in the sirtuin signaling pathway and the production of reactive oxygen species in both skeletal muscle and EFP, leading to insulin resistance, mitochondrial dysfunction, and inflammation. On the other hand, AE upregulated skeletal muscle proteins (NDUFB5, NDUFS2, NDUFS7, ETFD, FRDA, and MKNK1) that enhance mitochondrial function and insulin sensitivity. Additionally, the upregulation of LDHC and PRKACA and the downregulation of CTBP1 in EFP can promote the browning of white adipose tissue with the involvement of FNDC5/irisin in the canonical pathway. Our study provides insights into AE-induced molecular responses and may help further develop exercise-mimicking therapeutic targets.

## 1. Introduction

Obesity is known to be caused by a positive energy balance between energy intake and energy expenditure that leads to excessive fat accumulation in adipose tissue and ectopic tissues, including the liver, heart, and skeletal muscle [1]. In prolonged overnutrition, white adipocytes accumulate more lipids and develop cellular hypertrophy [2], whereas skeletal muscle can undergo fatty infiltration and inflammation [3]. Excessive lipid accumulation leads to the upregulation of proinflammatory pathways, the production of excess reactive oxygen species (ROS), and mitochondrial dysfunction in both adipose tissue and skeletal muscle [4]. Dysfunctional adipose tissue and skeletal muscle can lead to insulin resistance (IR) in obesity [5]. Insulin allows glucose to enter cells for protein and fatty acid synthesis as well as energy metabolism and binds to receptors primarily on adipose tissue and skeletal muscle [6]. IR is a pathological condition in which insulin-mediated glucose uptake is impaired in insulin-sensitive tissue and plays a pivotal role in the pathogenesis of obesity-related metabolic diseases, such as type 2 diabetes mellitus, fatty liver disease, and cardiovascular disease [7,8].

Irrefutable evidence affirms the importance of aerobic exercise (AE) in the management of obesity through profound adaptations in both skeletal muscle and white adipose tissue (WAT) [9]. Myokines released after exercise can alter protein regulation and trigger complex molecular reactions, including changes in inflammatory cytokines, fatty acid oxidation, oxidative phosphorylation, and so on [10]. For instance, irisin, a fragmented product of transmembrane fibronectin type III domain-containing protein 5 (FNDC5), is a myokine secreted by skeletal muscle in response to exercise and accounts for several exercise-mediated health benefits, such as inducing white adipose tissue browning and enhancing insulin sensitivity [11,12]. As exercise represents one of the most effective interventions for combating obesity, proteomic analysis may help to explore exercise-induced molecular responses and uncover novel exercise-mimicking therapeutic targets [13].

Despite the growing evidence of the benefits of AE in obese mice, comparative proteomic analyses of both skeletal muscle and WAT in response to AE are limited [14,15]. The use of a high-throughput comprehensive proteomic analysis provides a more in-depth examination of the underlying mechanisms behind the benefits of AE in obesity at the level of individual translational proteins [16]. By gaining a deeper understanding of these processes, we can better elucidate the molecular mechanisms that contribute to the protective effects of AE. In this context, we explore the impact of AE on proteomic changes in the skeletal muscle and epididymal white adipose tissue of high-fat-diet (HFD)-induced obese mice.

## 2. Results

### 2.1. Effect of Aerobic Exercise on Body Weight and the Morphology of Skeletal Muscle and Epididymal Fat Pad

**Figure 1:**
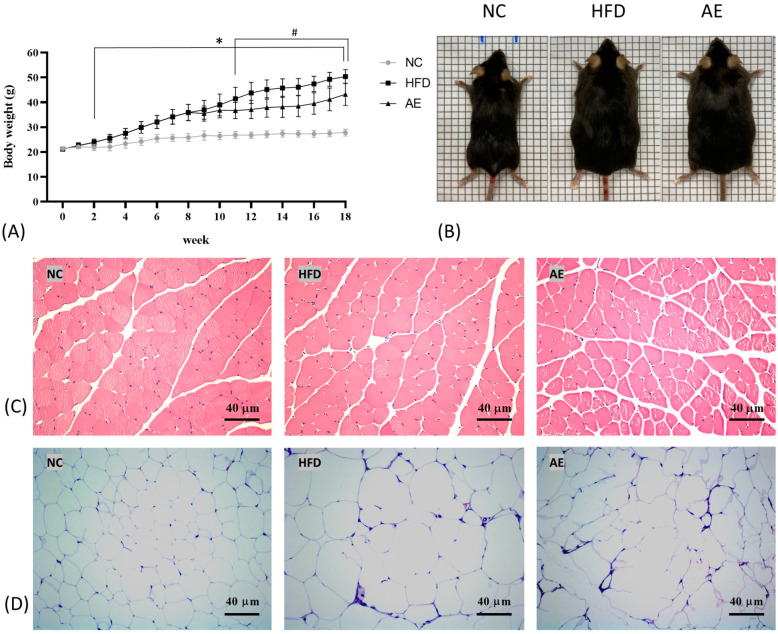
Effect of aerobic exercise on body weight (**A**), gross appearance of mice (**B**), morphology of skeletal muscle (**C**), and epididymal fat pad (**D**). Specimens were photographed using light microscopy (H&E stain, magnification: ×200, scale bar = 40 μm) (* *p* < 0.05 HFD vs. NC group, # *p* < 0.05 AE vs. HFD group).

The HFD induced significant weight gain from week 2 to week 8 in the HFD group compared with the normal control (NC) group (*p* < 0.05). By the end of 8 weeks of obesity induction, HFD-fed mice weighed 39% more than NC chow-fed mice (HFD: 35.87 ± 3.25 g, NC: 25.84 ± 1.57 g, *p* < 0.05). Body weight did not differ between the HFD and AE groups prior to the exercise intervention, but after 8 weeks of exercise training, AE significantly attenuated the weight gain compared to the sedentary HFD group (AE: 43.22 ± 4.50 g, HFD: 50.36 ± 2.79 g, *p* < 0.05) (Figure 1A). The gross appearances of mice in each group before sacrifice are shown in Figure 1B.

Hematoxylin and eosin (H&E) staining of skeletal muscle sections in the NC, HFD, and AE groups are shown in Figure 1C. The skeletal muscles were well preserved in morphology and structure, and the staining was well distributed in the NC group. The skeletal muscle from the HFD group exhibited some degenerative changes in muscle myofibers, whereas that from the NC group showed a normal architecture with dense myofibers. On the contrary, the AE group retained a normal architecture in the muscle myofibers, similar to that of the NC group. In addition, the H&E staining of epididymal fat pad (EFP) in the HFD group revealed white adipocyte hypertrophy after obesity induction. Compared to the HFD group, adipocytes were smaller in the AE group (Figure 1D).

### 2.2. Effect of Aerobic Exercise on Serum FNDC-5, Glucose, Insulin, and HOMA-IR Levels

**Figure 2:**
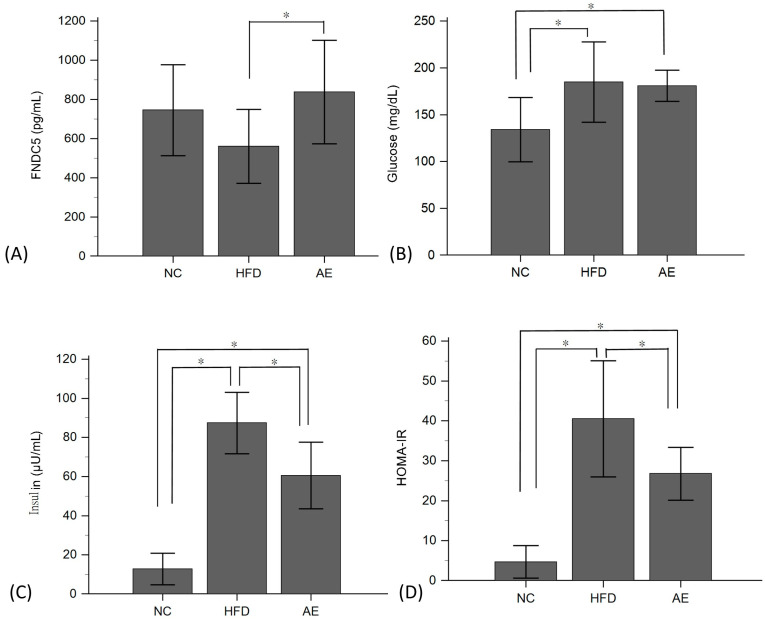
Effect of exercise on serum FNDC-5 (**A**), glucose (**B**), insulin (**C**), and HOMA-IR (**D**) levels (data are presented as means ± SDs; * *p* < 0.05 in ANOVA with Tukey’s multiple-comparison post hoc test).

We investigated the impact of AE on serum levels of FNDC-5, glucose, insulin, and the homeostasis model assessment of insulin resistance (HOMA-IR) (Figure 2). The results revealed that the serum FNDC-5 level in the AE group (837.36 ± 263 pg/mL) was significantly higher than that in the HFD group (560.66 ± 188 pg/mL) (*p* < 0.05). The HFD group exhibited significant increases in serum glucose, insulin, and HOMA-IR levels compared to the NC group (*p* < 0.05). However, 8 weeks of AE intervention significantly reduced serum insulin and HOMA-IR levels (*p* < 0.05), while there was no significant difference in serum glucose levels between the AE and HFD groups.

### 2.3. The Effect of HFD and AE on Skeletal Muscle Proteomic Changes

**Figure 3:**
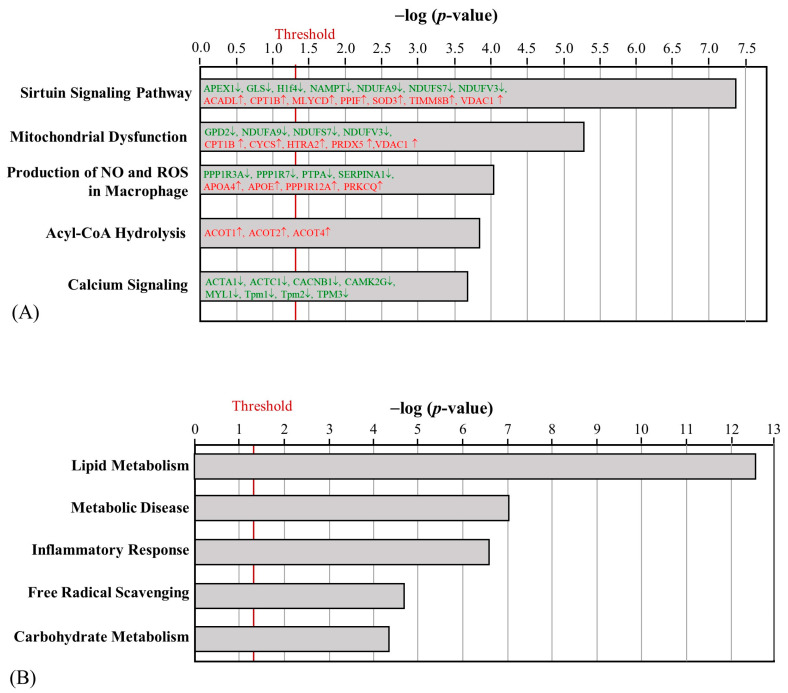
Effect of HFD on the canonical pathways (**A**) and biofunctions (**B**) regulated by skeletal muscle proteins between the NC and HFD groups. (The proteins with red letters were upregulated, whereas those with green letters were downregulated).

After HFD obesity induction, 81 and 99 proteins in the skeletal muscle were upregulated and downregulated, respectively (Supplementary Table S1). Three proteins were only detected in the HFD group, including Golgin subfamily A member 3 (GOGA3), Ras GTPase-activating-like protein IQGAP1 (IQGA1), and transmembrane protein 65 (TMM65). Significantly, the canonical pathways implicated changes in sirtuin signaling pathways, mitochondrial dysfunction, the production of nitric oxide (NO) and ROS in macrophages, Acyl-CoA hydrolysis, and calcium signaling (Figure 3A). Among the upregulated proteins, apolipoprotein E (APOE), apolipoprotein A4 (APOA4), and protein kinase C theta (PRKCQ) were expected to increase the production of ROS. On the other hand, the downregulation of NADH: ubiquinone oxidoreductase core subunit S7 (NDUFS7), subunit A9 (NDUFA9), and subunit V3 (NDUFV3) in mitochondrial complex I was associated with mitochondrial dysfunction. Regarding the biofunctions of differentially regulated skeletal muscle proteins, lipid metabolism and metabolic disease were significantly affected (Figure 3B). The upregulation of short-chain-specific acyl-CoA dehydrogenase (ACADS) and the downregulation of protein phosphatase 1 regulatory subunit 3A (PPR3A) can further promote IR.

**Figure 4:**
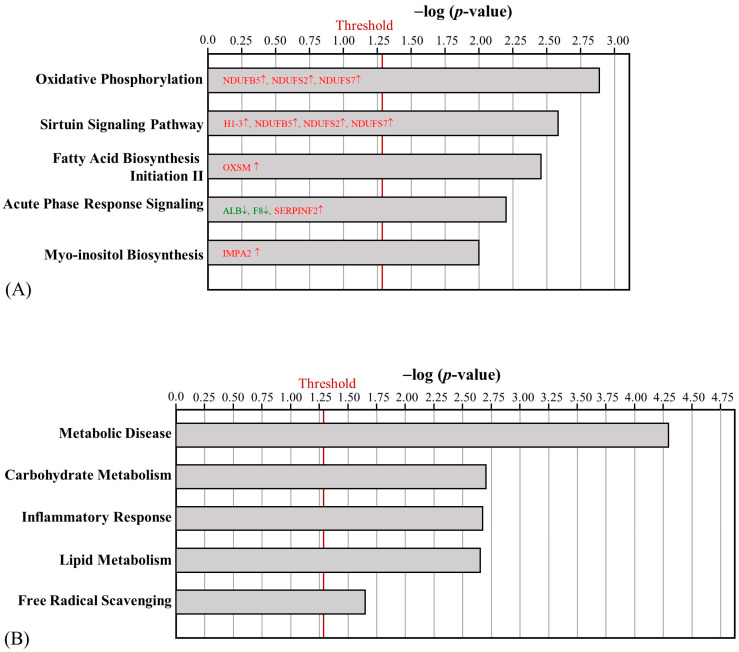
Effect of AE on the canonical pathways (**A**) and biofunctions (**B**) regulated by skeletal muscle proteins between the HFD and AE groups. (The proteins with red letters were upregulated, whereas those with green letters were downregulated).

The aerobic exercise intervention led to the identification of 47 differentially regulated proteins between the HFD and AE groups in mouse skeletal muscle, with 38 upregulated proteins and 9 downregulated proteins (Supplementary Table S2). MAP kinase-interacting serine/threonine-protein kinase 1 (MKNK1) was the only protein that was uniquely detected in the AE group. Several mitochondrial complex 1-related proteins that were downregulated by HFD, such as NDUFB5, NDUFS2, and NDUFS7, were upregulated after AE. Furthermore, the AE-altered proteins were involved in oxidative phosphorylation, the sirtuin signaling pathway, fatty acid biosynthesis initiation II, acute phase response signaling, and myo-inositol biosynthesis (Figure 4A). The differentially regulated skeletal muscle proteins were primarily involved in metabolic disease (Figure 4B). The upregulation of MKNK1, Frataxin (FRDA), and electron transfer flavoprotein-ubiquinone oxidoreductase (ETFD) can help decrease the risk of diabetes mellitus.

### 2.4. The Effect of HFD and AE on Epididymal Fat Pad Proteomic Changes

**Figure 5:**
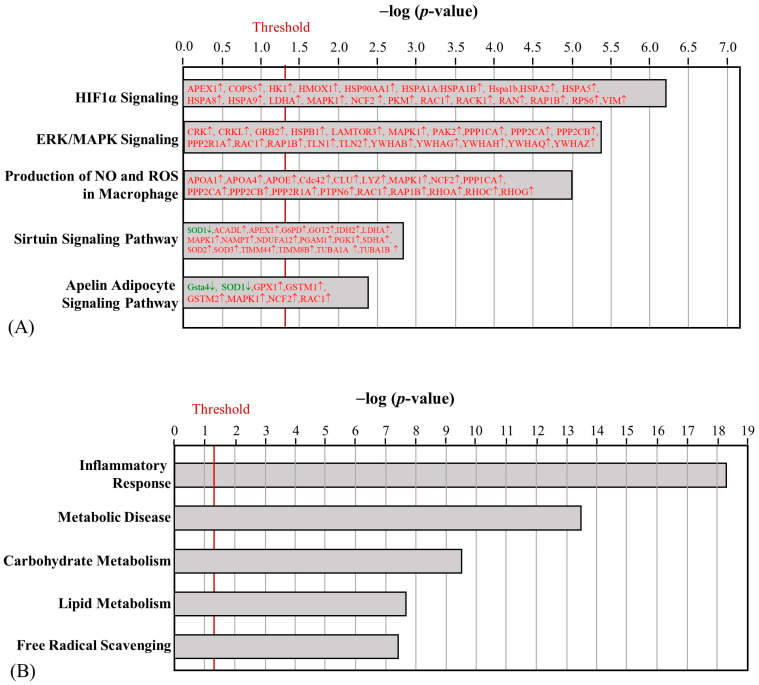
Effect of HFD on the canonical pathways (**A**) and biofunctions (**B**) regulated by EFP proteins between the NC and HFD groups. (The proteins with red letters were upregulated, whereas those with green letters were downregulated).

HFD obesity induction resulted in substantial differential protein regulation in EFP. A total of 714 differentially regulated proteins were found, with 698 upregulated proteins and 102 proteins that were exclusively detected in the HFD group (Supplementary Table S3). Among these uniquely detected proteins in the HFD group, the upregulation of mitogen-activated protein kinase 1 (MAPK1); neutrophil cytosol factor 2 (NCF2); protein tyrosine phosphatase non-receptor type 6 (PTPN6); and ras homolog family members A, C, and G (RHOA, RHOC, and RHOG) was associated with NO and ROS synthesis. The HFD-induced obesity-related proteins showed significant enrichment in the expected pathways, such as Hypoxia-Inducible Factor (HIF)1α signaling, extracellular-regulated kinase (ERK)/mitogen-activated protein kinase (MAPK) signaling, the production of NO and ROS in macrophages, the sirtuin signaling pathway, and the apelin adipocyte signaling pathway (Figure 5A). The most significant biofunction pathway was the inflammatory response (Figure 5B).

**Figure 6:**
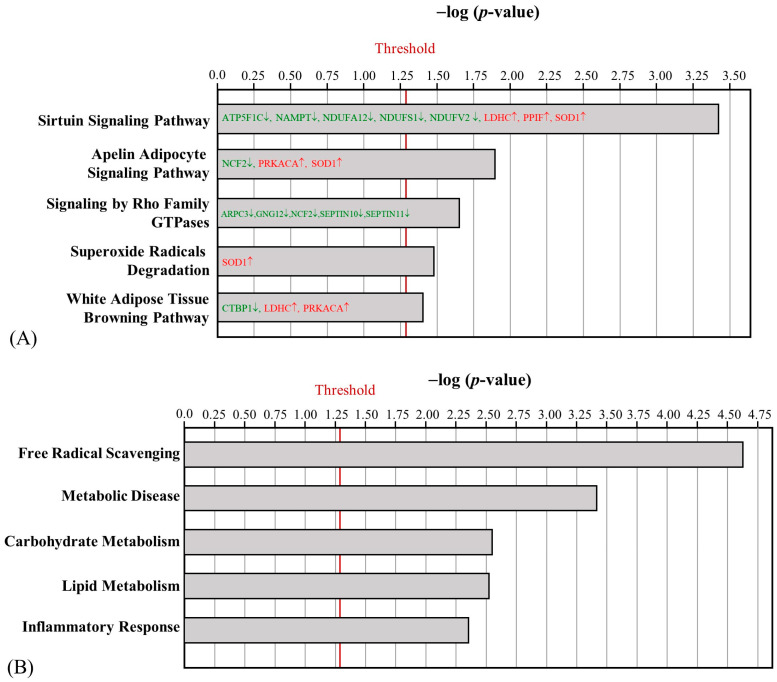
Effect of AE on the canonical pathways (**A**) and biofunctions (**B**) regulated by EFP proteins between the HFD and AE groups. (The proteins with red letters were upregulated, whereas those with green letters were downregulated).

Following aerobic exercise, 140 proteins were differentially regulated between the HFD and AE groups, with 29 upregulated proteins and 111 downregulated proteins (Supplementary Table S4). The differentially regulated proteins were significantly involved in the sirtuin signaling pathway, the apelin adipocyte signaling pathway, signaling by Rho family GTPases, superoxide radical degradation, and the white adipose tissue browning pathway (Figure 6A). It is noteworthy that the antioxidant enzyme superoxide dismutase 1 (SOD1), which was downregulated by HFD, was upregulated after AE. Additionally, white-adipose-tissue-browning-pathway-related proteins, lactate dehydrogenase C (LDHC), and protein kinase cAMP-activated catalytic subunit alpha (PRKACA) were significantly increased, and C-terminal binding protein 1 (CTBP1) was significantly decreased. Many differentially regulated EFP proteins were involved in free radical scavenging and metabolic disease (Figure 6B). The upregulation of peroxiredoxin-5 (PRDX5) as well as the downregulation of laminin subunit alpha-4 (LAMA4), long-chain fatty acid transport protein 1 (S27A1), and heat shock 70 kDa protein 12A (HS12A) can help counteract obesity-related IR.

## 3. Discussion

**Figure 7:**
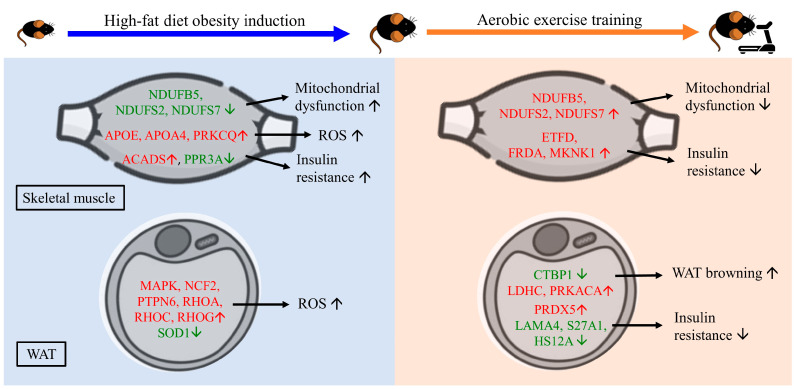
Graphical summary (WAT—white adipose tissue, ROS—reactive oxygen species) (The proteins with red letters were upregulated, whereas those with green letters were downregulated) (The black arrows pointing up and down represent predicted increases or decreases in biofunction, respectively.).

AE has been suggested as an effective strategy for managing obesity and related metabolic disorders through varied mechanisms, including the upregulation of antioxidant enzymes and the release of myokines, such as irisin, that facilitate crosstalk between skeletal muscle and adipose tissue [9]. To elucidate the impact of AE on the changes in skeletal muscle and EFP protein regulation in HFD-induced obese mice, a high-throughput quantification of the proteome and an ingenuity pathway analysis (IPA) were performed to understand the underlying canonical pathways and biofunctions. Our results show that 8 weeks of AE significantly attenuated body weight, increased serum FNDC5 levels, and improved HOMA-IR in HFD-induced obese mice. Furthermore, our study provides insight into the proteins regulated by HFD and AE in skeletal muscle and EFP and the underlying processes. HFD caused alterations in a subset of skeletal muscle and EFP proteins involved in IR, mitochondrial dysfunction, and inflammation. The skeletal muscle proteins (NDUFB5, NDUFS2, NDUFS7, ETFD, FRDA, and MKNK1) upregulated by AE can counteract IR and mitochondrial dysfunction. Moreover, the upregulation of LDHC and PRKACA in EFP can promote the browning of white adipose tissue, with FNDC5/irisin potentially involved in the underlying canonical pathway. A graphical summary is shown in Figure 7.

Our proteomic analysis found that HFD alters several IR-, mitochondrial-dysfunction-, stress-, and redox-related proteins in both skeletal muscle and EFP. Consistent with previous findings on the roles of ACADS [17] and PPR3A [18], we observed the upregulation of ACADS as well as the downregulation of PPR3A in the HFD group, which can contribute to IR. In addition, many of the significantly regulated proteins showed enrichment in the sirtuin signaling pathway and the production of NO and ROS in both the skeletal muscle and EFP proteomes. Sirtuins serve as sensors for the redox states of cells [19], and excess free fatty acid uptake in skeletal muscle can lead to impaired sirtuin-3 and further drive β-oxidation as well as oxidative stress [20].

AE has been demonstrated to improve insulin sensitivity in skeletal muscle [21] as well as reduce macrophage infiltration and inflammatory markers in WAT [22]. Previous proteomic studies investigating the effect of exercise on skeletal muscle have identified several differentially regulated proteins that ameliorate IR [23]. Our study further revealed a number of AE-regulated skeletal muscle proteins that can help improve IR, such as MKNK1 [24], FRDA [25], and ETFD [26]. In support of our findings, previous studies of transcriptome analyses of diabetes-prone and diabetes-resistant obese mice disclosed that the downregulation of MKNK1 in pancreatic islets is associated with diabetes [24]. In addition, our results show that ETFD was upregulated in skeletal muscle but downregulated in EFP after AE, indicating that the regulation of this protein may differ between tissues. We presume that this could be because ETFD is primarily located in the mitochondria [27], which makes it more easily upregulated in mitochondria-rich tissue, such as skeletal muscle, after AE. Our study provides novel insights into the role of AE in mitigating IR through protein regulation and highlights the importance of considering tissue-specific responses to exercise.

Increasing evidence supports the role of irisin in reducing IR by promoting glucose uptake in skeletal muscle and inducing the browning of WAT [28]. In our study, serum FNDC5 significantly increased in the AE group compared to the HFD group. The increased FNDC5 was presumably responsible for the canonical WAT browning pathway observed in the EFP proteome in the AE group. As shown in our IPA analysis, the downregulation of CTBP1 and the upregulation of LDHC and PRKACA in the AE group may contribute to FNDC5-induced WAT browning. We found that CTBP1 was differentially regulated in skeletal muscle and EFP after AE, with a greater fold change in EFP. Given CTBP1′s role as a transcriptional regulator of adipogenesis [29], we suggest that it should be taken into account in EFP. The downregulation of CTBP1 can repress WAT-specific genes and promote brown fat genes such as PPAR-gamma-coactivator-1α (PGC-1α) [29]. PRKACA can stimulate the phosphorylation of Creb proteins, further increase the expression of uncoupling protein 1 (UCP1) protein, and lead to WAT browning in the long run [30]. On the other hand, a study by Guilford et al. found a compensatory mechanism mediated by elevated FNDC5 to counteract HFD-induced obesity and IR [31]. However, that study was performed with 4 weeks of intervention, whereas our study was carried out for a total of 16 weeks, which suggests that chronic HFD feeding may diminish this protective effect and that AE can help elevate the FNDC5 level.

In addition, our IPA analysis revealed that the upregulation of SOD1 in the AE group can further enhance the antioxidant defense in EFP, possibly through the apelin adipocyte signaling pathway. In line with our findings, a previous study found that the overexpression of SOD1 protein reduces the generation of ROS [32]. As an adipocytokine secreted by and sensed by adipocytes, apelin is necessary for the maintenance of insulin sensitivity and tends to counteract diabetes mellitus [6]. The apelin adipocyte signaling pathway can dampen ROS generation, increase mitochondrial biogenesis, and promote the browning of WAT [33]. Furthermore, our study observed several differentially regulated EFP proteins in the AE group that can mitigate diet-induced obesity, such as the upregulated PRDX5 [34] as well as the downregulated LAMA4 [35], S27A1 [36], and HS12A [37], as reported in previous studies.

Our study has several limitations that warrant further investigation in future research. First, because our study mainly focused on the skeletal muscle and EFP proteomic changes in response to AE, we did not analyze the specific mRNA expression in the tissue. Integrating transcriptomic and proteomic analyses would provide a more comprehensive understanding of AE-mediated adaptation. Furthermore, in a label-free quantification proteomic analysis based on mass spectrometry (MS), it is possible that some proteins may be present but below the detection limit due to their low abundance or may be excluded by other high-abundance proteins, making them difficult to identify [38]. To overcome this limitation, our study only analyzed valid values with significant differences between groups. Additionally, we only examined EFP among all the WAT depots. We decided to explore the proteome of visceral WAT in EFP mainly because visceral fat is more harmful to metabolic health, and AE can significantly reduce visceral fat mass, but the underlying molecular mechanisms are not well understood [39]. Still, it would be beneficial to also analyze proteomic changes in other WAT depots, as visceral and subcutaneous WAT may exhibit different adaptations after AE.

In conclusion, our proteomic analysis demonstrates wide changes in proteins in AE-trained skeletal muscle and EFP, which provide insights into the molecular pathways of HFD-induced obesity mitigated by AE. HFD affects a wide range of proteins involved in IR, mitochondrial dysfunction, stress, and redox processes in both skeletal muscle and EFP. AE enhances insulin sensitivity and promotes proteins involved in free radical scavenging as well as the browning of WAT, with the potential involvement of FNDC5/irisin. MS-based proteomics offers great promise for finding AE-mimicking therapeutic targets to combat obesity.

## 4. Materials and Methods

### 4.1. Animal Experiment

**Figure 8:**
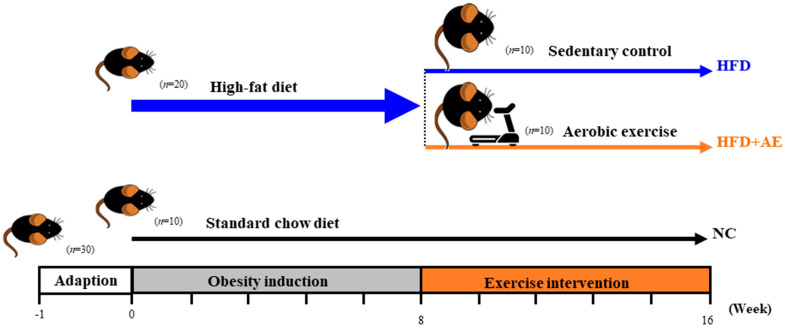
Experimental design (AE—aerobic exercise, HFD—high-fat diet, NC—normal control).

Five-week-old male C57BL/6J mice were obtained from Bio LASCO Taiwan (Taipei, Taiwan) and were housed in a controlled room at room temperature (22 ± 2 °C) on a 12 h dark/light cycle with water ad labium. After 1 week of acclimatization, the mice were initially divided into the NC group (*n* = 10) and HFD obesity induction group (*n* = 20). The NC group received a standard chow diet (13.5% of kcal from fat, 3.36 kcal/g, Laboratory rodent diet 5001; LabDiet, St. Louis, MO, USA), while the obesity induction group was fed with HFD (60% of kcal from fat, 5.24 kcal/g, Research Diets D12492; Research Diets, Inc., New Brunswick, NJ, USA) for up to 8 weeks. Body weight was measured twice a week. The experimental design is shown in Figure 8. The experiment was approved by the National Taiwan Sport University Institutional Animal Care and Use Committee (IACUC-11117) and was performed in accordance with the relevant guidelines and regulations.

### 4.2. Exercise Intervention Protocol

After 8 weeks of obesity induction, the HFD group was randomly subdivided into the sedentary HFD control and AE groups (n = 10 in each group). There was no significant weight difference between the groups before the exercise intervention. The exercise protocol was based on a previously published peer-reviewed study, with some small adaptations [40].

The AE group was subjected to running at a speed of 15 m/min for 40 min on a motor-driven treadmill with a 0% incline (model MK-680; Muromachi Kikai, Tokyo, Japan). Continuously adjustable electric shocks (0–500 V) from the grid at the end of the lane were used as stimuli to boost running. Mice were trained for 40 min per day 5 times per week, for a total of 8 weeks, and were continuously fed with HFD.

### 4.3. Blood Sample Analysis

At the end of the experiments, all animals were euthanized using 95% CO_2_ asphyxiation after 12 h of fasting. Blood samples were collected via cardiac puncture. The serum FNDC-5 and insulin levels were determined using commercially available enzyme-linked immunosorbent assay (ELISA) kits according to the manufacturer’s instructions (Mouse FNDC5 ELISA Kit, MBS2515765, MyBioSource, San Diego, CA, USA; Mercodia Mouse Insulin ELISA kit #10-1247-01).

The serum fasting blood glucose was measured using a Beckman DxC 800 autoanalyzer (Beckman Coulter, Brea, CA, USA). The HOMA-IR was calculated using the formula: HOMA-IR = [fasting insulin (μU/mL) × fasting glucose (mg/dL)]/405 [41].

### 4.4. Histology of Skeletal Muscle and Epididymal White Adipose Tissue

Following euthanasia, the EFP and gastrocnemius muscles were meticulously harvested, weighed, and stored at −80 °C for further analysis. Tissues were cut into small pieces and preserved in 10% formalin. The formalin-fixed samples were embedded in paraffin, sliced into 4 µm sections, and stained with H&E for microscopic observation using an Olympus BX-51 CCD camera (Tokyo, Japan).

### 4.5. Protein Extraction and Liquid Chromatography–Tandem Mass Spectrometry Analysis

Proteomic profiling was performed as previously described [42]. Frozen mouse tissues were homogenized using a lysis buffer containing phosphate-buffered saline with 0.1% Triton X-100, PhosSTOP™ phosphatase inhibitor, and cOmplete™ protease inhibitor cocktail (Roche Diagnostics Corporation, Indianapolis, IN, USA). Zirconium oxide beads (1.0 mm) were added in a ratio of 1:2:1 (tissue/lysis buffer/beads) by volume. Homogenization was performed at maximum speed for 30 s using a homogenizer (Next Advance Bullet Blender, BBX24B, Troy, NY, USA), followed by cooling on ice for 60 s. This process was repeated at least three times until the tissue was fully homogenized. The homogenized tissue fluid was then centrifuged at 15,000 rpm at 4 °C for 15 min, and the supernatant was used for protein concentration measurement (Pierce™ BCA Protein Assay Kit) or stored in a −80 °C ultralow-temperature freezer.

Protein digestion was performed using a SMART Digest kit, according to the manufacturer’s instructions. Briefly, the tissue samples were mixed with Digestion Buffer in a 1:3 ratio, and immobilized trypsin beads were added. The reaction was performed at 72 °C with shaking for 4 h. The supernatant was then collected using centrifugation, and dithiothreitol (final concentration: 1 mM) was added for a 30 min reaction at 53 °C, followed by the addition of iodoacetamide (final concentration: 5 mM) and incubation at room temperature in the dark for 30 min. After alkylation, the reaction products were cleaned up using solid-phase extraction.

Before MS analysis, the lyophilized peptides were resuspended in 0.1% formic acid and separated using a nanoflow liquid chromatography system (Vanquish neo). The samples were ionized using a nano-spray and analyzed using an Orbitrap Fusion Lumos tandem mass spectrometer in automated data-dependent acquisition analysis mode. The spectrometer performed a full scan of the positive ions in the range of *m*/*z* 300–1600 and captured ions based on the signal intensity within three seconds. If the charge state of the ions was 2+~6+, high-energy collision dissociation (HCD) was performed to collect MS/MS fragment ions. Raw MS and MS/MS data were collected for interpretation.

### 4.6. Mass Spectrometry Data Analysis

For protein identification, PEAKS 8.0 software was used to match the MS raw data against the reviewed UniProt Mouse database. The matching criteria included a peptide false discovery rate (FDR) of <0.01, a protein FDR of <0.05, a mass error of <15 ppm, and the identification of at least one unique peptide. For protein quantification, the spectral count method was used to calculate the number of peptide–spectrum matches (PSMs) for each protein. The total spectral count of each sample was used as the normalization factor when performing cross-sample comparisons. The average and standard deviation (SD) were then calculated for each group of samples, and a t-test was used to determine the significance of differences between groups. Proteins with *p*-values of <0.05 were considered to have significant differences.

### 4.7. Bioinformatic Analysis

The molecular function, biological process, and cellular component of each protein molecule were annotated using the Gene Ontology (GO) database. IPA software 2022 (QIAGEN Inc., Hilden, Germany, https://www.qiagenbioinformatics.com/products/ingenuitypathway-analysis, accessed on 3 October 2022) was utilized to perform a canonical pathway analysis [43]. The accession numbers and expression fold changes in the differentially regulated proteins were uploaded to the IPA software for biological function grouping and canonical pathway analysis. The significance (*p*-value of overlap) was calculated using Fisher’s exact test.

### 4.8. Statistical Analysis

The data were presented as means ± SDs and were analyzed using IBM SPSS 22 statistical software and MedCalc software (Version 20.211). Statistical significances were determined using a one-way analysis of variance (ANOVA) followed by the Duncan post hoc multiple range test. *p* < 0.05 was considered statistically significant.

## Data Availability

The data presented in this study are available on reasonable request from the corresponding author.

## Supplementary Materials

The supporting information can be downloaded at: https://www.mdpi.com/article/10.3390/ijms24065743/s1.

## References

[1] Heymsfield S.B., Wadden T.A. (2017). Mechanisms, Pathophysiology, and Management of Obesity. N. Engl. J. Med..

[2] Longo M., Zatterale F., Naderi J., Parrillo L., Formisano P., Raciti G.A., Beguinot F., Miele C. (2019). Adipose Tissue Dysfunction as Determinant of Obesity-Associated Metabolic Complications. Int. J. Mol. Sci..

[3] Pedersen B.K., Febbraio M.A. (2012). Muscles, exercise and obesity: Skeletal muscle as a secretory organ. Nat. Rev. Endocrinol..

[4] van der Kolk B.W., Saari S., Lovric A., Arif M., Alvarez M., Ko A., Miao Z., Sahebekhtiari N., Muniandy M., Heinonen S. (2021). Molecular pathways behind acquired obesity: Adipose tissue and skeletal muscle multiomics in monozygotic twin pairs discordant for BMI. Cell Rep. Med..

[5] Shulman G.I. (2014). Ectopic fat in insulin resistance, dyslipidemia, and cardiometabolic disease. N. Engl. J. Med..

[6] Li M., Chi X., Wang Y., Setrerrahmane S., Xie W., Xu H. (2022). Trends in insulin resistance: Insights into mechanisms and therapeutic strategy. Signal Transduct. Target. Ther..

[7] Müller T.D., Blüher M., Tschöp M.H., DiMarchi R.D. (2022). Anti-obesity drug discovery: Advances and challenges. Nat. Rev. Drug Discov..

[8] Ahmed B., Sultana R., Greene M.W. (2021). Adipose tissue and insulin resistance in obese. Biomed. Pharmacother..

[9] Chow L.S., Gerszten R.E., Taylor J.M., Pedersen B.K., van Praag H., Trappe S., Febbraio M.A., Galis Z.S., Gao Y., Haus J.M. (2022). Exerkines in health, resilience and disease. Nat. Rev. Endocrinol..

[10] Contrepois K., Wu S., Moneghetti K.J., Hornburg D., Ahadi S., Tsai M.S., Metwally A.A., Wei E., Lee-McMullen B., Quijada J.V. (2020). Molecular Choreography of Acute Exercise. Cell.

[11] Boström P., Wu J., Jedrychowski M.P., Korde A., Ye L., Lo J.C., Rasbach K.A., Boström E.A., Choi J.H., Long J.Z. (2012). A PGC1-α-dependent myokine that drives brown-fat-like development of white fat and thermogenesis. Nature.

[12] Rabiee F., Lachinani L., Ghaedi S., Nasr-Esfahani M.H., Megraw T.L., Ghaedi K. (2020). New insights into the cellular activities of Fndc5/Irisin and its signaling pathways. Cell Biosci..

[13] Deshmukh A.S. (2016). Proteomics of Skeletal Muscle: Focus on Insulin Resistance and Exercise Biology. Proteomes.

[14] Deshmukh A.S., Murgia M., Nagaraj N., Treebak J.T., Cox J., Mann M. (2015). Deep proteomics of mouse skeletal muscle enables quantitation of protein isoforms, metabolic pathways, and transcription factors. Mol. Cell Proteom..

[15] Tang X., Li J., Zhao W.G., Sun H., Guo Z., Jing L., She Z., Yuan T., Liu S.N., Liu Q. (2019). Comprehensive map and functional annotation of the mouse white adipose tissue proteome. PeerJ.

[16] Chen C., Hou J., Tanner J.J., Cheng J. (2020). Bioinformatics Methods for Mass Spectrometry-Based Proteomics Data Analysis. Int. J. Mol. Sci..

[17] Chen Y., Chen J., Zhang C., Yang S., Zhang X., Liu Y., Su Z. (2019). Deficiency in the short-chain acyl-CoA dehydrogenase protects mice against diet-induced obesity and insulin resistance. FASEB J..

[18] Delibegovic M., Armstrong C.G., Dobbie L., Watt P.W., Smith A.J., Cohen P.T. (2003). Disruption of the striated muscle glycogen targeting subunit PPP1R3A of protein phosphatase 1 leads to increased weight gain, fat deposition, and development of insulin resistance. Diabetes.

[19] Singh C.K., Chhabra G., Ndiaye M.A., Garcia-Peterson L.M., Mack N.J., Ahmad N. (2018). The Role of Sirtuins in Antioxidant and Redox Signaling. Antioxid. Redox Signal..

[20] Cortés-Rojo C., Vargas-Vargas M.A., Olmos-Orizaba B.E., Rodríguez-Orozco A.R., Calderón-Cortés E. (2020). Interplay between NADH oxidation by complex I, glutathione redox state and sirtuin-3, and its role in the development of insulin resistance. Biochim. Biophys. Acta (BBA)—Mol. Basis Dis..

[21] Li N., Shi H., Guo Q., Gan Y., Zhang Y., Jia J., Zhang L., Zhou Y. (2022). Aerobic Exercise Prevents Chronic Inflammation and Insulin Resistance in Skeletal Muscle of High-Fat Diet Mice. Nutrients.

[22] Kawanishi N., Niihara H., Mizokami T., Yada K., Suzuki K. (2015). Exercise training attenuates neutrophil infiltration and elastase expression in adipose tissue of high-fat-diet-induced obese mice. Physiol. Rep..

[23] Yuan H., Niu Y., Liu X., Yang F., Niu W., Fu L. (2013). Proteomic Analysis of Skeletal Muscle in Insulin-Resistant Mice: Response to 6-Week Aerobic Exercise. PLoS ONE.

[24] Chan J.Y., Bensellam M., Lin R.C.Y., Liang C., Lee K., Jonas J.-C., Laybutt D.R. (2021). Transcriptome analysis of islets from diabetes-resistant and diabetes-prone obese mice reveals novel gene regulatory networks involved in beta-cell compensation and failure. FASEB J..

[25] Tatsch E., De Carvalho J.A.M., Bollick Y.S., Duarte T., Duarte M., Vaucher R.A., Premaor M.O., Comim F.V., Moresco R.N. (2020). Low frataxin mRNA expression is associated with inflammation and oxidative stress in patients with type 2 diabetes. Diabetes Metab. Res. Rev..

[26] Jain P., Vig S., Datta M., Jindel D., Mathur A.K., Mathur S.K., Sharma A. (2013). Systems biology approach reveals genome to phenome correlation in type 2 diabetes. PLoS ONE.

[27] Henriques B.J., Katrine Jentoft Olsen R., Gomes C.M., Bross P. (2021). Electron transfer flavoprotein and its role in mitochondrial energy metabolism in health and disease. Gene.

[28] Chen J.Q., Huang Y.Y., Gusdon A.M., Qu S. (2015). Irisin: A new molecular marker and target in metabolic disorder. Lipids Health Dis..

[29] Lo K.A., Sun L. (2013). Turning WAT into BAT: A review on regulators controlling the browning of white adipocytes. BioSci. Rep..

[30] Dickson L.M., Gandhi S., Layden B.T., Cohen R.N., Wicksteed B. (2016). Protein kinase A induces UCP1 expression in specific adipose depots to increase energy expenditure and improve metabolic health. Am. J. Physiol.-Regul. Integr. Comp. Physiol..

[31] Guilford B.L., Parson J.C., Grote C.W., Vick S.N., Ryals J.M., Wright D.E. (2017). Increased FNDC5 is associated with insulin resistance in high fat-fed mice. Physiol. Rep..

[32] Zhao M.M., Xu M.J., Cai Y., Zhao G., Guan Y., Kong W., Tang C., Wang X. (2011). Mitochondrial reactive oxygen species promote p65 nuclear translocation mediating high-phosphate-induced vascular calcification in vitro and in vivo. Kidney Int..

[33] Palmer E.S., Irwin N., O’Harte F.P. (2022). Potential Therapeutic Role for Apelin and Related Peptides in Diabetes: An Update. Clin. Med. Insights Endocrinol. Diabetes.

[34] Kim M.H., Park S.J., Kim J.H., Seong J.B., Kim K.M., Woo H.A., Lee D.S. (2018). Peroxiredoxin 5 regulates adipogenesis-attenuating oxidative stress in obese mouse models induced by a high-fat diet. Free Radic. Biol. Med..

[35] Vaicik M.K., Blagajcevic A., Ye H., Morse M.C., Yang F., Goddi A., Brey E.M., Cohen R.N. (2018). The Absence of Laminin α4 in Male Mice Results in Enhanced Energy Expenditure and Increased Beige Subcutaneous Adipose Tissue. Endocrinology.

[36] Wu Q., Ortegon A.M., Tsang B., Doege H., Feingold K.R., Stahl A. (2006). FATP1 is an insulin-sensitive fatty acid transporter involved in diet-induced obesity. Mol. Cell Biol..

[37] Zhang X., Chen X., Qi T., Kong Q., Cheng H., Cao X., Li Y., Li C., Liu L., Ding Z. (2019). HSPA12A is required for adipocyte differentiation and diet-induced obesity through a positive feedback regulation with PPAR?. Cell Death Differ..

[38] Schork K., Podwojski K., Turewicz M., Stephan C., Eisenacher M. (2021). Important Issues in Planning a Proteomics Experiment: Statistical Considerations of Quantitative Proteomic Data. Methods Mol. Biol..

[39] Lehnig A.C., Stanford K.I. (2018). Exercise-induced adaptations to white and brown adipose tissue. J. Exp. Biol..

[40] Xu X., Ying Z., Cai M., Xu Z., Li Y., Jiang S.Y., Tzan K., Wang A., Parthasarathy S., He G. (2011). Exercise ameliorates high-fat diet-induced metabolic and vascular dysfunction, and increases adipocyte progenitor cell population in brown adipose tissue. Am. J. Physiol.-Regul. Integr. Comp. Physiol..

[41] Matthews D.R., Hosker J.P., Rudenski A.S., Naylor B.A., Treacher D.F., Turner R.C. (1985). Homeostasis model assessment: Insulin resistance and β-cell function from fasting plasma glucose and insulin concentrations in man. Diabetologia.

[42] Chou T.J., Lu C.W., Liao C.C., Chiang C.H., Huang C.C., Huang K.C. (2022). Ovariectomy Interferes with Proteomes of Brown Adipose Tissue in Rats. Int. J. Med. Sci..

[43] Krämer A., Green J., Pollard J., Tugendreich S. (2014). Causal analysis approaches in Ingenuity Pathway Analysis. Bioinformatics.

